# Impact of Internal Medicine Subspecialty Consultations on Length of Stay: A Pilot Retrospective Cohort Study

**DOI:** 10.7759/cureus.102730

**Published:** 2026-01-31

**Authors:** Ashley L Fang, Christina J Dietz

**Affiliations:** 1 Department of Internal Medicine, Division of Hospital Medicine, The Ohio State University Wexner Medical Center, Columbus, USA

**Keywords:** inpatients, internal medicine, internal medicine subspecialties, length of stay, referral and consultation

## Abstract

Objective

For many years, the average length of stay for inpatient hospitalization has been steadily increasing, with hospitalists citing many potential reasons for the increase. Some of the commonly known reasons are time spent waiting for consultant recommendations, procedure delays, and diagnostic testing delays. The objective of this study was to preliminarily examine the relationship between the number of internal medicine subspecialty consultations requested and inpatient length of stay (LOS) on a single hospital medicine service.

Design, setting, and participants

This pilot study was a retrospective cohort study at one large tertiary academic medical center. Included participants were adults who were admitted to one hospital medicine service line from January 1, 2022, to December 31, 2022. The main outcome was the impact of the total number of internal medicine subspecialty consultations on the length of stay (LOS) index (observed LOS divided by expected LOS). Secondary outcome measures were between-group baseline demographic differences, primary admission diagnoses, and discharge admission diagnoses.

Results

Patients with internal medicine subspecialty consults had a significantly higher expected LOS (6.10 days vs 4.73; p<0.0001), observed LOS (7.67 days vs 5.04; p<0.0001), and LOS index (1.17 vs 1.03; p<0.0001). The most common admission and discharge diagnosis was COVID-19. In the univariate and multivariable analyses, demographic variables were not significantly associated with the LOS index. After controlling for covariates, the LOS index increased by 0.3178 for each additional consult. Non-diabetes endocrinology, palliative care, and rheumatology were the internal medicine subspecialty consultations associated with the greatest increase in the LOS index.

Conclusion

Patients in the consult group experienced a 1.5-day longer overall length of stay, with each additional consultation resulting in a 32% increase in the ratio of observed to expected LOS.

## Introduction

Prolonged hospital admissions in the United States remain a significant burden for patients and hospital systems. From 2019 to 2022, the average length of stay for inpatients increased from 4.3 days to 5.3 days, representing a nearly 18% increase [1]. Since payors reimburse based on a patient’s diagnosis, hospitals are not reimbursed for additional hospital time, regardless of the level of care required during that period [2]. This leaves hospitals needing to cover the additional expenditure. The American Hospital Association estimated that underpayments from Medicare and Medicaid totaled more than $130 billion in the year 2023 alone [3]. These shortfalls continue to grow, with an average annual increase of approximately 14% between 2019 and 2023 [3].

In addition to the ever-growing cost of care, prolonged hospital stays are associated with a variety of hospital-acquired infections, such as *Clostridium difficile* infections (CDI) [4], catheter-associated urinary tract infections (CAUTI) [5], and central line-associated bloodstream infections (CLABSI) [6,7]. Estimates from the Agency for Healthcare Research and Quality demonstrated an increase of up to $94,000 per inpatient hospitalization for CLABSI alone in 2015 [8]. More recent data estimates a 4-year total cost addition of $23.4M and an additional 4810 excess hospital days across the United States due to the three most common hospital-acquired infections (CLABSI, CAUTI, and surgical site infections), with the increased cost driven primarily by CLABSI and CAUTI [9].

Additionally, prolonged hospital stay has been identified as a risk factor for falls in elderly patients [10] and a predictor of mortality in patients admitted for esophageal hemorrhage [11] and acute heart failure [12]. A number of causes of prolonged length of stay on medical services have been identified, with several studies utilizing a survey-based approach to detect trends [13,14]. Hospitalists identified that time spent waiting for consultant recommendation and procedure delays are significant contributing factors to prolonged hospitalization [13], with one study demonstrating an average delay of 1.3 to 1.8 days due to consultant factors [14].

Although several studies have evaluated the impact of single specialty consultation on inpatient length of stay, we are unaware of any studies that assess the impact of the number of specialties consulted on inpatient length of stay. In this study, our goal was to examine the relationship between the number of internal medicine subspecialty consultations requested and the inpatient length of stay on a general internal medicine service at a large tertiary medical center.

## Materials and methods

Study design, setting, and patients 

This is a retrospective cohort study design. This pilot study examined data from a one-year period at a large tertiary academic medical center in central Ohio. This study was deemed exempt by the organization’s institutional review board.

The study included all patients who were 18 years or older and admitted to the largest hospital medicine inpatient service between January 1, 2022, and December 31, 2022. The patient level of care for patients admitted to this medical service is medical-surgical or progressive care.

Patients who were admitted to another medical service, different hospitals within the medical center, had multiple discharge locations, or were discharged to court/law enforcement were excluded from the sample.

Study variables 

The primary variable of interest was the length of stay index, which is defined as the observed length of stay for the patient divided by the expected length of stay. The expected length of stay is a metric that is calculated based on a person’s Medicare severity diagnosis-related group (MS-DRG) codes. The observed length of stay is the amount of time the patient remained in the hospital.

The primary outcome variable was the total number of consultations, defined as the sum of the 13 internal medicine subspecialty categories potentially consulted for the patient’s index admission. The 13 internal medicine subspecialty consultation categories are for this study are nephrology, infectious disease, diabetes-related endocrinology, cardiology, hematology, oncology, gastroenterology, pulmonology, rheumatology, immunology, palliative care, hepatology, and non-diabetes-related endocrinology. These were selected based on the subspecialties within the academic medical center’s Department of Internal Medicine and how those respective subspecialties are commonly consulted for this level of care.

Demographic variables include age at admission, biological sex, race, ethnicity, and body mass index (BMI). BMI was categorized into six primary categories (underweight, normal weight, overweight, Class I obesity, Class II obesity, Class III obesity), plus a missing category. Level of care discusses the intensity of care that patients receive while they are hospitalized. On this medical service line, the two levels of care provided are medical-surgical and progressive care. Discharge location is where the patient is released after hospitalization. For patients receiving care on this medical service line, the possible discharge locations range from home care to expired.

Case mix index (CMI) is a measure that defines the diversity, complexity, and severity of care required for a patient and is used for hospital reimbursement, resource allocation, and performance measurement. Over time, this measure has become a surrogate for patient-level disease severity. This measure was selected to represent the patient’s disease severity over other measures, such as the Charlson Comorbidity Index (CCI), due to CMI focusing on the patient’s present acuity and the limited literature for CCI around younger, less severely ill patients [15-17]. The admission’s primary ICD-10 code was selected to represent the top medical reason a patient was admitted to the service line.

Statistical analysis 

Continuous outcomes are reported as medians and ranges. Categorial outcomes are reported as numbers and percentages. Between-group comparisons were done with either the Mann-Whitney U test or the 𝜒2 test. Simple and multivariable linear regressions were conducted to examine factors associated with the index length of stay while controlling for age, race, sex, categorized BMI, and CMI. Race was recategorized from six to four groups due to insufficient sample size in several categories. Discharge location was recategorized from 21 to 9 groups to reduce the degrees of freedom. Statistical significance was set at p <0.05. All analyses were conducted using SAS, v. 9.4, software (SAS Institute, Cary, NC).

## Results

Patient characteristics 

A total of 5,114 patients were included in this study, with 3,248 (63.5%) having consultations from other internal medicine subspecialties (Table 1). The average age of participants in both groups was approximately 60 years old (p=0.5651), with BMIs approximately 28.0 kg/m^2^ ( p=0.8290). Both groups had slightly more men than women (X2=1.3283;p=0.2491) and the majority of the participants were white (consult=73.2%, no consult=71.76%) or black (consult=21.2%, no consult=22.45%) (p=0.6810). Additionally, the majority of participants were not Hispanic or Latino (consult=96.6%, no consult=96.52%) (p=0.8811).

**Table 1:** Characteristics of patients with and without consults * denotes significance at p<0.05 ** Mann-Whitney U test (U-value) was used for continuous variables and chi-square test (X^2^ value) for categorical variables.

Demographics	Patients with Consults (N=3248)	Patients without Consults (N=1866)	Test Statistic**	p-value
Age: Median (Range)	60 (19-109)	61 (18 - 100)	0.5753	0.5651
BMI: Median (Range)	27.7 (11.30 - 98.60)	28.0 (11.20 - 81.60)	0.216	0.8290
Case Mix Index: Median (Range)	1.60 (0.55 - 20.24)	1.10 (0.38 - 20.24)	-18.7377	<0.0001*
Expected Length of Stay: Median (Range)	6.10 (1.62 - 151.07)	4.73 (1.34 - 55.25)	-15.9814	<0.0001*
Observed Length of Stay: Median (Range)	7.67 (0.54 - 251.13)	5.04 (0.21 - 175.21)	-16.0169	<0.0001*
Length of Stay Index: Median (Range)	1.17 (0.09 - 17.30)	1.03 (0.05 - 46.12)	-5.4521	<0.0001*
Sex: N (%)	-	-	1.3283	0.2491
Female	1594 (49.1)	919 (49.25)	-	-
Male	1654 (50.9)	947 (50.75)	-	-
Race: N (%)	-	-	1.5055	0.6810
White	2376 (73.2)	1339 (71.76)	-	-
Black or African American	687 (21.2)	419 (22.45)	-	-
Asian	44 (1.35)	23 (1.23)	-	-
Other/Prefer Not to Answer	141 (4.34)	85 (4.56)	-	-
Ethnicity: N (%)	-	-	0.2532	0.8811
Hispanic or Latino	77 (2.37)	47 (2.52)	-	-
Not Hispanic or Latino	3136 (96.6)	1801 (96.52)	-	-
Prefer Not to Answer / Other / Unknown	35 (1.08)	18 (0.96)	-	-
BMI Categories: N (%)	-	-	73.3907	<0.0001*
Missing	461 (14.2)	434 (23.26)	-	-
Underweight (BMI: Less than 18.5)	170 (5.23)	82 (4.39)	-	-
Normal Weight (BMI: 18.5 - 24.9)	760 (23.4)	406 (21.76)	-	-
Overweight (BMI: 25.0 - 29.9)	745 (22.9)	349 (18.70)	-	-
Obesity Class I (BMI: 30.0 - 34.9)	457 (14.1)	267 (14.31)	-	-
Obesity Class II (BMI: 35.0 - 39.9)	293 (9.02)	155 (8.31)	-	-
Obesity Class III (BMI: Greater than 40.0)	362 (11.1)	173 (9.27)	-	-
Discharge Location: N (%)	-	-	195.8218	<0.0001*
Acute Care Group	39 (1.20)	32 (1.71)	-	-
Psych Care Group	24 (0.74)	116 (6.22)	-	-
AMA Care Group	72 (2.22)	58 (3.11)	-	-
Home Care Group	2221 (68.4)	1208 (64.74)	-	-
SNF Care Group	607 (18.7)	362 (19.40)	-	-
Rehab Care Group	70 (2.16)	53 (2.84)	-	-
Long-Term Care Group	50 (1.54)	15 (0.80)	-	-
Hospice Facility Care Group	54 (1.66)	10 (0.54)	-	-
Expired Group	111 (3.42)	12 (0.64)	-	-
Consults: Median (Range)	1 (1 - 8)	-	-	N/A
Number of Consults: N (%)	-	-	-	N/A
1 consult	1802 (55.5)	-	-	-
2 consults	848 (26.1)	-	-	-
3 consults	366 (11.3)	-	-	-
4 consults	139 (4.28)	-	-	-
5 consults	57 (1.75)	-	-	-
6 consults	23 (0.71)	-	-	-
7 consults	9 (0.28)	-	-	-
8 consults	4 (0.12)	-	-	-
Service Consulted: N (%)	-	-	-	N/A
Nephrology	952 (29.3)	-	-	-
Infectious Disease	1258 (38.7)	-	-	-
Endocrinology: Diabetes Mellitus	274 (8.44)	-	-	-
Cardiology	710 (21.9)	-	-	-
Hematology	227 (6.99)	-	-	-
Oncology	90 (2.77)	-	-	-
Gastroenterology	945 (29.1)	-	-	-
Pulmonology	395 (12.2)	-	-	-
Rheumatology	131 (4.03)	-	-	-
Immunology	41 (1.26)	-	-	-
Palliative Care	230 (7.08)	-	-	-
Hepatology	310 (9.54)	-	-	-
Endocrinology: Non-Diabetes Mellitus	107 (3.29)	-	-	-

Baseline sociodemographic and clinical characteristics 

Compared to patients without consults, patients with consults had a significantly higher CMI (1.60 vs 1.10; p<0.0001) (Table 1). The expected length of stay for the consult group was 6.10 days compared to 4.73 days (p<0.0001). The median observed length of stay for the consult group was 7.67 days, whereas the median for the patients without consults was 5.04 days (p<0.0001). The median length of stay index for the consult group was 1.17 compared to 1.03 for patients without consults (p<0.0001). After categorizing BMI into the six categories plus missing, almost half of the patients had normal or overweight BMIs, whereas for the patients without consults, almost half had missing or overweight BMIs (p<0.0001). There was a statistically significant difference between the locations where patients were discharged. Patients in both groups were commonly discharged to home care, but the patients without consults had a higher percentage discharged to psychiatric care or left against medical advice (p<0.0001).

For the patients with consults, the median number of consults was 1, with 55.5% of patients in this group having 1 consult. The most commonly consulted subspecialties were infectious disease (38.7%), nephrology (29.3%), gastroenterology (29.1%), and cardiology (21.9%). The least consulted subspecialties were rheumatology (4.03%), non-diabetes mellitus endocrinology (3.29%), oncology (2.77%), and immunology (1.26%).

Admission and discharge diagnoses 

For the patients with consults, the common primary admissions ICD-10 codes were for COVID-19, unspecified organ sepsis, and unspecified acute kidney failure (Table 2). For patients without consultations, the common primary admission ICD-10 codes were COVID-19, unspecified altered mental status, and unspecified cerebral infarction. When looking at the top 10 primary admission ICD-10 codes for the groups, both groups had COVID-19, unspecified encephalopathy, unspecified acute kidney failure, and shortness of breath.

**Table 2:** Top-10 admission and discharge diagnoses for patients

Patients with Consults	N (%)
Primary Admission Diagnosis ICD-10 Code	
U07.1: COVID-19	91 (2.80)
A41.9: Sepsis, unspecified organism	88 (2.71)
N17.9: Acute kidney failure, unspecified	79 (2.43)
R10.9: Unspecified abdominal pain	70 (2.16)
K70.31: Alcoholic cirrhosis of the liver with ascites	41 (1.26)
R06.02: Shortness of breath	37 (1.14)
T86.19: Other complication of kidney transplant	37 (1.14)
G93.40: Encephalopathy, unspecified	36 (1.11)
J96.01: Acute respiratory failure with hypoxia	32 (0.99)
J96.21: Acute and chronic respiratory failure with hypoxia	32 (0.99)
Primary Discharge Diagnosis ICD-10 Code	-
U07.1: COVID-19	105 (3.23)
A41.9: Sepsis, unspecified organism	90 (2.77)
T86.19: Other complication of kidney transplant	72 (2.22)
K70.31: Alcoholic cirrhosis of the liver with ascites	55 (1.69)
N17.9: Acute kidney failure, unspecified	49 (1.51)
I13.0: Hypertensive heart and chronic kidney disease with heart failure and stage 1 through stage 4 chronic kidney disease, or unspecified chronic kidney disease	48 (1.48)
I11.0: Hypertensive heart disease with heart failure	41 (1.26)
E11.69: Type 2 diabetes mellitus with other specified complication	34 (1.05)
A41.89: Other specified sepsis	30 (0.92)
I13.2: Hypertensive heart and chronic kidney disease with heart failure and with stage 5 chronic kidney disease, or end-stage renal disease	29 (0.89)
Patients Without Consults	N (%)
Primary Admission Diagnosis ICD-10 Code	-
U07.1: COVID-19	73 (3.91)
R41.82: Altered mental status, unspecified	43 (2.30)
I63.9: Cerebral infarction, unspecified	37 (1.98)
R53.1: Weakness	37 (1.98)
F10.239: Alcohol dependence with withdrawal, unspecified	36 (1.93)
R56.9: Unspecified convulsions	36 (1.93)
G93.40: Encephalopathy, unspecified	35 (1.88)
R45.851: Suicidal ideations	33 (1.77)
N17.9: Acute kidney failure, unspecified	27 (1.45)
R06.02: Shortness of breath	25 (1.34)
Primary Discharge Diagnosis ICD-10 Code	-
U07.1: COVID-19	96 (5.14)
F10.239: Alcohol dependence with withdrawal, unspecified	39 (2.09)
N39.0: Urinary tract infection, site not specified	38 (2.04)
A41.9: Sepsis, unspecified organism	31 (1.66)
N17.9: Acute kidney failure, unspecified	25 (1.34)
G93.40: Encephalopathy, unspecified	24 (1.29)
J18.9: Pneumonia, unspecified organism	24 (1.29)
G40.909: Epilepsy, unspecified, not intractable, without status epilepticus	23 (1.23)
G93.41: Metabolic encephalopathy	22 (1.18)
G92.8: Other toxic encephalopathy	21 (1.13)

For the patients with consults, the common discharge diagnosis ICD-10 codes were COVID-19, unspecified organism sepsis, and other complications of kidney transplant. For patients without consultations, the common discharge diagnosis ICD-10 codes were COVID-19, unspecified alcohol dependence with withdrawal, and site-not-specified urinary tract infection. When comparing the top 10 discharge diagnosis ICD-10 codes for the groups, both groups had COVID-19, unspecified organism sepsis, and unspecified acute kidney failure.

Factors impacting the LOS index 

**Figure 1:**
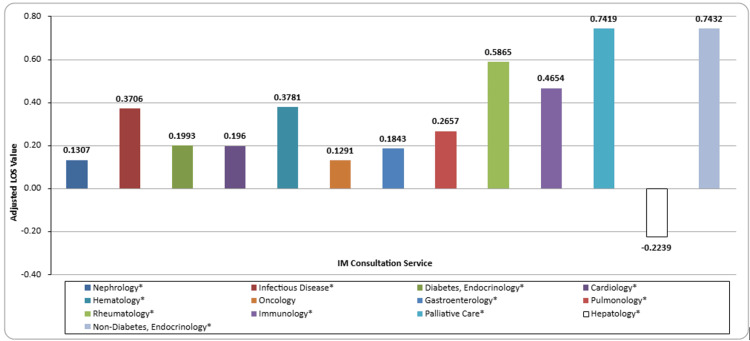
Adjusted length of stay values by the internal medicine consultation service * denotes significance at p<0.05

Table 3 shows the results for the univariate and multivariable linear regressions. Many of the variables were found to be significantly associated with the length of stay index. The total number of consults was found to be significantly positively associated with increased length of stay index (p<0.001). Similarly, CMI was found to be associated with an increased length of stay index (p=0.006). Age, race, sex, ethnicity, and level of care were found not to be associated with the LOS index. All categories of BMI, except obesity class III, were found to be significantly negatively associated with the LOS index, with the categories of missing (p<0.0001) and underweight (p=0.0006) having the largest negative associations. Four of the six discharge locations were significantly associated with the LOS index. For individuals who were discharged to long-term care, they had a 1.0010 increased length of stay index compared to those discharged to home care (p<0.0001). Individuals who left against medical advice had a 0.3867 decrease in the LOS index (p=0.0169). All consultation subspecialties, except oncology, were found to be associated with an increased length of stay index (Figure 1). Non-diabetes endocrinology (p<0.0001), palliative care (p<0.0001), and rheumatology (p<0.0001) were the three subspecialty services consulted that resulted in the largest significant increases in length of stay index. Hepatology was the only subspecialty that was negatively associated with the length of stay index (p=0.0120).

**Table 3:** Relationships between length of stay index and associated factors, controlling for covariates Ref: reference *Significant at P<0.05 **Adjusted for age, race, sex, categorized BMI, and case mix index (CMI)

	Unadjusted B Coefficient	95% CI	P	Adjusted B Coefficient**	95% CI	P
Total Number of Consults	0.3150	0.2726 to 0.3574	<0.0001*	0.3178	0.2730 to 0.3625	<0.0001*
CMI	0.0428	0.0183 to 0.0673	0.0006*	0.0400	0.0154 to 0.0645	0.0014*
Age	0.0006	-0.0021 to 0.0034	0.6569	0.0006	-0.0021 to 0.0034	0.6487
Race						
White	Ref	Ref	Ref	Ref	Ref	Ref
Black or African American	0.0613	-0.0550 to 0.1776	0.3013	0.0690	-0.0476 to 0.1856	0.2461
Asian	0.1956	-0.2129 to 0.6041	0.3480	0.2037	-0.2031 to 0.6105	0.3263
Other/Prefer Not to Answer	0.1593	-0.0734 to 0.3921	0.1796	0.1952	-0.0377 to 0.4281	0.1005
Sex						
Male	Ref	Ref	Ref	Ref	Ref	Ref
Female	-0.0076	-0.1019 to 0.0866	0.8737	0.0022	-0.0923 to 0.0967	0.9634
Ethnicity						
Not Hispanic or Latino	Ref	Ref	Ref	Ref	Ref	Ref
Hispanic or Latino	0.1668	-0.1429 to 0.4765	0.2910	0.0884	-0.2802 to 0.4570	0.6383
Other/Prefer Not to Answer	-0.3081	-0.7644 to 0.1482	0.1857	-0.4174	-0.8883 to 0.0535	0.0823
BMI Categories						
Normal (BMI: 18.5 - 24.9)	Ref	Ref	Ref	Ref	Ref	Ref
Underweight (BMI:	-0.3965	-0.6232 to -0.1698	0.0006*	-0.4023	-0.6290 to -0.1756	0.0005*
Overweight (BMI: 25.0 - 29.9)	-0.1547	-0.2924 to -0.0169	0.0278*	-0.1499	-0.2878 to -0.0120	0.0331*
Obesity Class I (BMI: 30.0 - 34.9)	-0.2298	-0.3880 to -0.0716	0.0044*	-0.2219	-0.3801 to -0.0637	0.0060*
Obesity Class II (BMI: 35.0 - 39.9)	-0.2103	-0.3941 to -0.0266	0.0249*	-0.2019	-0.3857 to -0.0182	0.0313*
Obesity Class III (BMI: > 40.0)	-0.0170	-0.1876 to 0.1537	0.8457	-0.0074	-0.1791 to 0.1643	0.9325
Missing	-0.4215	-0.5793 to -0.2638	<0.0001*	-0.4040	-0.5622 to -0.2458	<0.0001*
Discharge Location						
Home	Ref	Ref	Ref	Ref	Ref	Ref
Acute Care	0.1359	-0.2920 to 0.5638	0.5336	0.1462	-0.2811 to 0.5734	0.5024
Psych	0.2811	-0.2626 to 0.8247	0.3108	0.2684	-0.2738 to 0.8107	0.3318
AMA	-0.3867	-0.7039 to -0.0695	0.0169*	-0.3994	-0.7183 to -0.0804	0.0141*
SNF	0.3961	0.2748 to 0.5175	<0.0001*	0.4130	0.2862 to 0.5398	<0.0001*
Rehab	0.6439	0.3223 to 0.9655	<0.0001*	0.6248	0.3012 to 0.9484	0.0002*
Long-Term Care	1.0010	0.6307 to 1.3884	<0.0001*	1.0023	0.6113 to 1.3932	<0.0001*
Hospice	0.6004	0.2359 to 0.9653	0.0013*	0.6025	0.2332 to 0.9718	0.0014*
Expired	0.3945	0.1368 to 0.6521	0.0027*	0.4158	0.1540 to 0.6776	0.0019*
Level of Care						
Med/Surg	Ref	Ref	Ref	Ref	Ref	Ref
Progressive Care	-0.0616	-0.1936 to 0.0703	0.3600	-0.1071	-0.2400 to 0.0257	0.1139
Nephrology Consult	0.1658	0.0624 to 0.2691	0.0017*	0.1307	0.0259 to 0.2355	0.0145*
Infectious Disease Consult	0.3825	0.2866 to 0.4783	<0.0001*	0.3706	0.2729 to 0.4684	<0.0001*
Diabetes, Endocrinology Consult	0.1851	0.0156 to 0.3545	0.0323*	0.1993	0.0292 to 0.3694	0.0217*
Cardiology Consult	0.2327	0.1189 to 0.3463	<0.0001*	0.1960	0.0792 to 0.3129	0.0010*
Hematology Consult	0.4079	0.2226 to 0.5922	<0.0001*	0.3781	0.1943 to 0.5618	<0.0001*
Oncology Consult	0.1650	-0.1220 to 0.4520	0.2598	0.1291	-0.1588 to 0.4170	0.3793
Gastroenterology Consult	0.1759	0.0723 to 0.2794	0.0009*	0.1843	0.0807 to 0.2879	0.0005*
Pulmonology Consult	0.2972	0.1534 to 0.4410	<0.0001*	0.2657	0.1195 to 0.4120	0.0004*
Rheumatology Consult	0.5947	0.3560 to 0.8333	<0.0001*	0.5865	0.3482 to 0.8247	<0.0001*
Immunology Consult	0.5132	0.0915 to 0.9349	0.0171*	0.4654	0.0450 to 0.8858	0.0300*
Palliative Care Consult	0.7834	0.6017 to 0.9651	<0.0001*	0.7419	0.5571 to 0.9267	<0.0001*
Hepatology Consult	-0.2054	-0.3656 to -0.0452	0.0120*	-0.2239	-0.3845 to -0.0633	0.0063*
Non-Diabetes, Endocrinology Consult	0.7432	0.4804 to 1.0059	<0.0001*	0.7432	0.4811 to 1.0053	<0.0001*

After controlling for age, race, sex, CMI, and categorized BMI, for each additional consult added for a patient, the length of stay index increases by 0.3178 (p<0.0001). Similar to the univariate analysis, age, race, sex, ethnicity, and level of care were found not to be associated with the LOS index. Associations for categorization remained consistent after controlling for covariates. The associations for rheumatology (p<0.0001), immunology (p=0.0300), palliative care (p<0.0001), and hepatology (p=0.0063) were negatively attenuated, but remained significant. The non-diabetes endocrinology consult remained unchanged after controlling for covariates.

## Discussion

In this single-center, retrospective, pilot study, patients in the group with internal medicine subspecialty consultations experienced a 1.5-day longer overall length of stay. For the group without these consultations, the median observed LOS was similar to the median expected LOS (5.04 days and 4.73 days, respectively). However, for the group with internal medicine subspecialty consultations, the difference between median expected LOS and median observed LOS (6.10 days and 7.67 days, respectively) is much larger. These findings indicate that when internal medicine subspecialists were not consulted, the hospitalists managing these patients discharged them within the expected time frame.

After controlling for potential covariates, each additional consultation resulted in an increase of 0.3178, which is a 32% increase in the ratio of observed to expected LOS. Although variability was observed between the various subspecialties, all studied subspecialties were associated with a statistically significant increase in the LOS index, with the exception of hepatology and medical oncology. Interestingly, hepatology consultation resulted in a 0.2239 (22%) decrease in the length of stay index, which is likely due to the observed increased expected LOS in this medically complex population. While patients with medical oncology consultations trended toward a higher LOS index, this finding was not statistically significant, likely due to a combination of low total consults for this service and a tendency at this institution to discharge patients with suspected malignancy and pending studies (i.e., pathology results and imaging studies) to complete their evaluation as an outpatient.

Procedure wait times have been previously cited as a consultation factor contributing to increased LOS [13]; however, we did not observe a more profound LOS impact associated with procedure-heavy specialties, such as gastroenterology and cardiology. Instead, we observed a more robust increase in the LOS index when non-procedural specialties, such as non-diabetes endocrinology, palliative care, rheumatology, immunology, and infectious diseases, were consulted. The most likely explanation for this observation relates to laboratory study wait time or goals of care planning [18,19]. For example, in cases where non-diabetes endocrinology, rheumatology, and immunology are consulted, complex multi-step testing may be ordered, and many highly specialized laboratory studies require shipping time and processing at an outside facility [18,20]. Infectious disease consultants must await culture data before making final recommendations, with cultures often taking several days for finalization, likely contributing to increased LOS [18,21]. Additionally, in many cases requiring these subspecialists, consultations are often delayed while the patient undergoes a complex diagnostic evaluation. Finally, many of these subspecialty services have fewer clinicians available to address a new consultation, while others (such as infectious diseases) are consulted at a much higher volume, resulting in longer wait times for consultation recommendations [22].

Unsurprisingly, we observed an increased LOS index for patients discharged to long-term care, inpatient rehabilitation, hospice, and skilled nursing facilities. This is consistent with observations from prior studies [13,23] and has been cited by the American Hospital Association as a significant contributor to prolonged LOS [3]. Several factors are likely involved in this observation, including a lack of robust discharge planning services, awaiting prior authorization from various payors, and bed availability at in-network post-discharge facilities [3]. To address this factor, hospitals should consider further developing their discharge planning services and workflows to encourage more timely transitions to post-acute care facilities.

In this pilot study, we evaluated a hospital system with more than 28,000 annual patient discharges and a hospitalist cohort service line with more than 6,500 annual patient discharges at a large academic institution. Although some of the individual internal medicine subspecialties included in this study may not be available for consultation at smaller hospitals, this study speaks to a systemic issue within hospital medicine that directly affects length of stay metrics. As hospital systems across the country face worsening financial shortfalls [2,3], the impact of increased inpatient subspecialty consultation cannot be overlooked [22]. Hospitalists should feel empowered to manage conditions independently that are commonly within their scope of practice and should carefully consider the necessity of inpatient subspecialty consults. However, hospitalists must remain cautious, given previous observations correlating increased length of stay with delays in appropriate specialist consultation [14]. An additional factor that should be considered is whether a consultation is needed during the current inpatient hospitalization or if the patient would be better served with an outpatient subspecialty referral. Outpatient referrals may be particularly beneficial for the cohort of patients who are medically stable for discharge but are awaiting laboratory results. A more robust outpatient network may allow for earlier discharge and appropriate post-discharge follow-up, where laboratory and radiologic studies are addressed.

While this study has a number of strengths, it does have some limitations. The primary limitation is that this is a pilot retrospective cohort study examining data from a single medical service line at a single institution, which limits the generalizability of the results. Another limitation is the use of CMI as a marker of disease severity. While this is a common surrogate measure, this metric was not created for that purpose and hinges on appropriate clinical documentation. However, it is well-known that clinical documentation is a common area for improvement for many organizations. This study did not control for time to consult, and the role it plays in prolonging the LOS. Future studies should include time to consult as a confounding factor in analyses. Lastly, the LOS index is calculated based on an individual’s expected versus observed LOS. Similar to CMI, the expected length of stay is dependent upon the accurate documentation of relevant information during that patient’s admission.

An additional limitation of this study is the decision to include only internal medicine subspecialty consultations, rather than all available consultants. While including other types of consultant services, such as neurology, may impact the study results, the specific focus on internal medicine subspecialty consults does provide several benefits. One benefit to including only internal medicine subspecialty consults is broader generalizability to institutions where non-internal medicine consultant services may be limited. Another benefit is that many of the conditions managed by internal medicine subspecialty consultants are within the scope of practice for internal medicine-trained hospitalists, whereas conditions managed by non-internal medicine subspecialty consultants are not within scope for hospitalists [24].

## Conclusions

This study found that inpatient internal medicine subspecialty consultations are associated with an increased LOS index for patients admitted to a hospitalist-managed medical service, despite controlling for demographic factors and illness severity. Hospitalists should carefully consider the medical necessity of inpatient consultations and balance this with the risks associated with increased length of stay. Additional studies are needed to examine the effect of inpatient subspecialty consultation across a longer timeframe, non-internal medicine subspecialties, inpatient teaching services, and following the modification of other factors associated with increased LOS (i.e., improved discharge planning initiatives).
